# The Relationship Between Uncertainty and Affect

**DOI:** 10.3389/fpsyg.2019.02504

**Published:** 2019-11-12

**Authors:** Eric C. Anderson, R. Nicholas Carleton, Michael Diefenbach, Paul K. J. Han

**Affiliations:** ^1^Center for Outcomes Research and Evaluation, Maine Medical Center Research Institute, Portland, ME, United States; ^2^Department of Medicine, Tufts University Medical Center, Boston, MA, United States; ^3^Department of Psychology, University of Regina, Regina, SK, Canada; ^4^Departments of Medicine, Urology, and Psychiatry, Northwell Health, New York, NY, United States

**Keywords:** uncertainty, affect, uncertainty tolerance, emotion, risk, simulation

## Abstract

Uncertainty and affect are fundamental and interrelated aspects of the human condition. Uncertainty is often associated with negative affect, but in some circumstances, it is associated with positive affect. In this article, we review different explanations for the varying relationship between uncertainty and affect. We identify “mental simulation” as a key process that links uncertainty to affective states. We suggest that people have a propensity to simulate negative outcomes, which result in a propensity toward negative affective responses to uncertainty. We also propose the existence of several important moderators of this process, including context and individual differences such as uncertainty tolerance, as well as emotion regulation strategies. Finally, we highlight important knowledge gaps and promising areas for future research, both empirical and conceptual, to further elucidate the relationship between uncertainty and affect.

## Introduction

Uncertainty and affective feelings are both fundamental aspects of human life. People are uncertain about the weather, how long they will live, and how other human beings will act in a given situation. People experience affective feelings (e.g., anger, anxiety, and pleasure) related to traffic, medical diagnoses, and social interactions. Uncertainty and affect also appear to be closely linked to each other. People typically find uncertainty to be aversive (Carleton, 2016b) and are willing to pay to reduce uncertainty (Lovallo and Kahneman, 2000); however, in some circumstances, people seem to find uncertainty attractive and seek out uncertainty-inducing activities—e.g., reading mystery novels (Zillmann, 1996), watching sports (Knobloch-Westerwick et al., 2009), or gambling. In fact, removing uncertainty from these activities seems to reduce enjoyment (e.g., movie or story spoilers). The reasons for these differences in people’s affective responses to uncertainty, however, are not well understood. Investigators from various psychological disciplines have offered some explanations; however, there is not a single, widely accepted, unifying theory accounting for the relationship between uncertainty and affect.

In this article, we explore this relationship further by reviewing important insights from existing theoretical accounts. Our objective is not to conduct a systematic review of all existing theories with potential relevance to this topic, and but rather to explore some of the more prominent theories that have focused explicitly on the experience of uncertainty, affect, and emotion. Our overarching aim is to synthesize common themes and ideas raised by these theories, and to identify potential mechanisms that might link uncertainty and affect. We acknowledge the existence of promising theoretical and empirical work in related fields—e.g., computational neuroscience—but leave the task of integrating this work for future analyses. We will show that existing theories of uncertainty, affect, and emotion suggest the importance of the psychological process of “mental simulation” as a key mediating factor in their relationship, and suggest potentially fruitful directions for future research to advance our understanding of these phenomena.

## The Nature of Uncertainty

An essential initial task of any analysis such as this is to establish a useful working definition of the term “uncertainty.” Despite the large volume of scholarship on uncertainty by psychologists and other social scientists, this term has often been either not explicitly defined or else defined in varying and often inconsistent ways. The Merriam-Webster dictionary defines uncertainty as “the state of being uncertain” and uses a plethora of terms to describe what it means to be uncertain: indefinite, indeterminate, not certain to occur, problematical, not reliable, untrustworthy, not known beyond doubt, dubious, doubtful, not clearly identified or defined, not constant, variable, and fitful.

The Merriam-Webster dictionary definition highlights an important area of agreement on the nature of uncertainty—the notion that uncertainty is fundamentally a mental state, a subjective, cognitive experience of human beings rather than a feature of the objective, material world. The specific focus of this experience, furthermore, is ignorance—i.e., the lack of knowledge. Importantly, uncertainty is not equivalent to mere ignorance; rather, uncertainty is the conscious awareness, or subjective *experience* of ignorance. It is a higher-order metacognition representing a particular kind of explicit knowledge*—*an acknowledgment of *what* one does not know, but also *that* one does not know. Smithson (1989) has used the term “meta-ignorance” to describe this state of knowing that one is ignorant, but we believe the term “uncertainty” better distinguishes this higher-order state from plain, unconscious ignorance (the state of not knowing that one is ignorant).

We believe the distinction between uncertainty and ignorance is critical to the phenomenology of uncertainty. Unless a person has some awareness of their ignorance, it is unlikely to influence their thoughts, feelings, or actions. We acknowledge that there are varying levels of conscious awareness, however, and that the awareness of ignorance may occur at a preconscious or unconscious level. For example, perceptual awareness of numerous other kinds of stimuli exists without higher-level conscious awareness; individuals constantly register and form inferences from perceptual data through unconscious, automatic processes. Cognitive scientist Andy Clark has argued that perceptual uncertainty is largely reduced by unconscious automatic processes, and that human beings can be characterized as being engaged in a continuous act of “surfing uncertainty” (Clark, 2015). In the same vein, dual-process theories of cognition distinguish unconscious, automatic “System 1” processes, from conscious, deliberate “System 2” processes, and a large body of empirical evidence has demonstrated that System 1 processes exert significant influence on judgment and decision making (Gigerenzer and Goldstein, 1996; Greene et al., 2001; Masicampo and Baumeister, 2008; Kahneman, 2011; Rand et al., 2012).

One can thus argue that uncertainty can exist below full conscious awareness. However, the problem then becomes one of defining what full conscious awareness means, and what minimal level of consciousness of ignorance is necessary for uncertainty to exist as an experientially and psychologically consequential state. We cannot resolve this problem here, but simply contend that the conceptual boundaries separating conscious and unconscious awareness are fuzzy and debatable (see later section on “Conceptual Issues”), and exactly where the boundaries should be set depends on one’s objectives. Our primary objective is to understand the relationship between uncertainty and affect as conscious, consequential experiences—i.e., as states that are fully manifest in one’s awareness. This interest is largely driven by our practical concern with understanding of what uncertainties people identify as being problematic in their lives, how these uncertainties influence medical decision making and mental health (Han et al., 2011), and what strategies people use to tolerate uncertainty (Han, 2013; Hillen et al., 2017; Strout et al., 2018). For these reasons, we focus on uncertainties that lie squarely within people’s conscious awareness, although we acknowledge that some uncertainties do not.

Researchers have distinguished three different sources of uncertainty (Han et al., 2011). The first source of uncertainty, probability (also commonly referred to as risk), arises from the randomness or indeterminacy of the future. The second source, ambiguity, arises from limitations in the reliability, credibility, or adequacy of probability (risk) information (Ellsberg, 1961). The final source, complexity, arises from features of available information that make it difficult to comprehend, such as multiple possible causes or outcomes. Because our goal is to tie together broad themes relating uncertainty to affect, we do not highlight the considerable work which has focused on the individual sources of uncertainty (Ellsberg, 1961; Tversky and Kahneman, 1981; Camerer and Weber, 1992; Rabin and Thaler, 2001; Siegrist et al., 2005; Zhang et al., 2014; Duttle and Inukai, 2015; Kovářík et al., 2016).

## The Nature of Affect and Emotions

Another essential initial task of our analysis is to establish useful working definitions of “affect” and “emotions.” Both terms signify distinct mental states that are discrete and also distinguishable from moods. Moods, such as depression or mania, are thought to represent more diffuse states that are longer in duration and not necessarily caused by one particular stimulus or event. In this article, we will primarily use the term “affect” because it is inclusive enough to include the broad range of findings described here, but the more specific term “emotion” when discussing research that focuses on those discrete states.

*Affect* has often been used as an umbrella term that signifies feelings of pleasure or discomfort, arousal, stress, emotion, and mood. Used in a more specific way, however, affect is thought to represent a feature of mental states comprised of two dimensions: valence, which ranges from pleasant to unpleasant; and arousal, which ranges from activated to deactivated (Russell and Barrett, 1999; Russell, 2003; Barrett, 2006). Thought theorists typically posit pleasant (positive affect) and unpleasant (negative affect) are poles on a single scale, others argue they are distinct processes that can be disassociated (Cacioppo et al., 2012). At any given moment, a person’s affective state can be described in terms of some combination of valence and arousal, and these feelings are thought to be an important component of a unified conscious experience (Wundt, 1998; Barrett and Bliss-Moreau, 2009).

Emotions, as distinguished from affect, are mental states whose essential nature has been an ongoing focus of lively debate (Barrett et al., 2007a). Discrete emotions are particular psychological states like disgust, guilt, or happiness. These emotional states have affective features: fear and anger are both typically unpleasant, high-arousal states, but the subjective experiences of fear and anger are not the same, and they have different causes and consequences. Importantly, the cause of an emotional response could be a physical stimulus, like seeing a bear on the trail ahead. Alternatively, however, the stimulus could also be a self-generated mental state, such as imagining being chased, or remembering being chased by a bear.

### The Relationship Between Uncertainty and Affect: General Theories of Affect and Emotion

Having offered working definitions of both uncertainty and affect, we now turn to the central question of interest of the current analysis: the relationship between these two mental states. Foundational theories of affect and emotion offer some insights on the psychological mechanisms connecting uncertainty and affect.

The basic “Modal Model” of emotions (Barrett et al., 2007b; Gross, 2014) is a widely accepted theory that offers a simplified, but useful, commonsense starting point for thinking about the process by which emotions are generated. The “modal model” suggests that emotions are generated by (1) a situation, that is (2) attended to, and then (3) appraised, which creates (4) an emotional response (Gross, 2014). Emotions are typically thought to represent a coordinated yet flexible multisystem response including changes to the autonomic nervous system, facial expressions, non-verbal behaviors, actions, and subjective feelings. These multisystem responses can then lead persons to change their focus of attention and to modify the situation, which creates a new cycle of emotion generation. For example, when Jill is walking alone at night (1), she might spot and attend to a shadowy alley (2). When approaching the alley, Jill might surveil the alley to look for evidence of threat. If a suspicious figure is spotted and appraised as a potential threat (3), Jill’s heart rate might quicken, muscles tense, and she might detour around the alley and have the subjective experience of feeling afraid (4). The action of detouring might lead to meeting an old friend, which would then trigger a new emotion generation process. On the other hand, the same situation might be experienced very differently if some contextual features are different. For instance, if Jill is looking for a secret nightclub (1), she might attend to an alley looking for signs of the club (2). Someone spotted in the alley might be appraised as a potential resource—someone who could give directions (3). This might lead to Jill’s muscles relaxing, approaching to ask for directions, and the subjective experience of feeling relief or excitement (4).

Appraisal theories of emotion are another important class of theories that specify a direct relationship between uncertainty and emotions (for a summary, see Moors et al., 2013). At their core, appraisal theories hold that emotions are adaptive processes that reflect appraisals of features of the environment that are significant for the organism’s survival and well-being. While appraisals can be conscious, rule-based processes, they are more often automatic, associations that match patterns in the environment to appraisals. Important appraisal variables include goal relevance, goal congruence, coping potential, and agency. Uncertainty vs. certainty about goals and outcomes is another appraisal dimension proposed by many appraisal theorists (see Moors et al., 2013). Therefore, the appraised uncertainty of a situation is fundamentally linked to the experience of different emotions. For example, the emotion “sadness” might be associated with certainty about a negative outcome. Consider a patient first learning she has been diagnosed with cancer. If she is convinced that treatment will not control her cancer (i.e., expresses high certainty about the lack of treatment efficacy), she might experience profound sadness. However, if she appraises the situation as less certain (e.g., the cancer might not progress or treatment might be effective), she might have a different emotional response. Thus, according to appraisal theories, the perceived uncertainty vs. certainty of a situation is a fundamental determinant of what specific emotion processes are elicited in that situation (Moors et al., 2013).

One strength of appraisal theories is that they are compatible with people having different responses to the same situation. If two people differ in their appraisal of a situation’s certainty, goal congruence, controllability, or other appraisals, their corresponding emotional response will also differ. Appraisal theories also generally assume that the same stimuli will not always cause same emotions because the intervening appraisals might differ. However, the same appraisals should consistently cause the same emotions (Moors et al., 2013).

The ability to imagine oneself in different situations, or simulate different perspectives, is thought to help people solve problems and make decisions (for discussion see, Taylor et al., 1998). Current theories link thoughts about the future (prospection), remembrances of the past (memory), and understandings of the viewpoint of others (theory of mind) to a common brain network, which includes the frontal and medial temporal-parietal lobes (Buckner and Carroll, 2007). People use these cognitive processes to imagine possible future events and to imagine their resulting affective responses to create “affective forecasts” (Wilson and Gilbert, 2005). There are biases associated with affective forecasts (Gilbert et al., 1998), but there is also evidence that people use their affective forecasts to make decisions in many life domains (e.g., medical care; Ferrer et al., 2015). As we will discuss later, the capacity to simulate different outcomes might be a key link between uncertainty and affect.

#### Emotion Regulation

**Figure 1:**
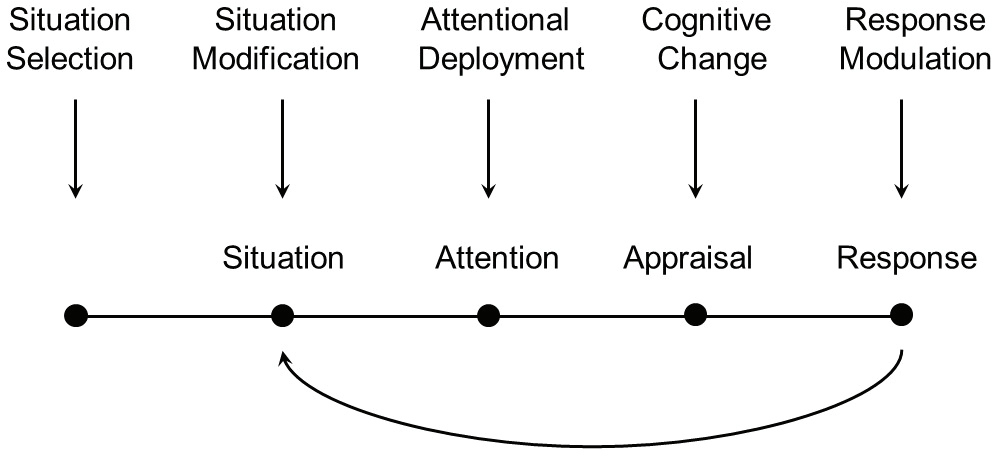
The process model of emotion regulation, from Gross (2014). Reprinted with permission of Guilford Press.

Affect and emotions can be controlled and regulated using various strategies. According to the process model (for review see, Gross, 2014), emotion regulation strategies can be usefully mapped onto different time points corresponding to different regulatory opportunities: situation selection, situation modification, attentional deployment, cognitive change, and response modulation (see Figure 1). For example, even before an emotion is generated, people can sometimes select the situation they are in (e.g., watching a comedy vs. horror movie). Next, people can modify the situation they are in (e.g., during a scary scene, turn the lights on, or mute the sound). Next, people can control what stimulus they attend to in the situation (e.g., closing eyes). People can also use cognitive processes to change how they think about the situation or their ability to meet the challenges posed by the situation (e.g., reappraising the stimulus as a movie—not as reality). Finally, people can attempt to modify their responses to the emotional episode (e.g., forcing a smile or taking deep breaths). These different regulation strategies align with the different components of emotion generation described above in the modal model of emotions.

#### Affect and Emotion Drive Action

Most contemporary theories construe affect and emotions as adaptive processes that serve the key function of preparing the body for action. Specifically, affective responses prepare the body for *possible actions* using predictions about what physiological resources will be needed (i.e., allostasis; Sterling, 2012). This is important because in uncertain situations the affective response will drive physiological responses based on predictions. For instance, at the sight of a bear, the brain makes a quick prediction that fighting or fleeing might be needed, which triggers increased heart rate as the body prepares for action. This example points to a common theme: affect and emotions are theorized to represent fast and frugal ways of representing the world and making decisions quickly—often in uncertain, complex situations where conscious, deliberative reasoning is impractical (see the evaluative space model as one example; Cacioppo et al., 2012).

Several theories construe affect as a type of information processing and cognition (Duncan and Barrett, 2007). For example, the affect-as-information theory (Schwarz and Clore, 1983) suggests people can use their own transient affective states as information to make judgments. In similar fashion, the affect heuristic theory (Slovic et al., 2007) suggests that mental representations of objects, events, and options are associated with affective tags. When making a decision, people consider the pool of affectively tagged information, which provides a quick gist impression of the options. Similarly, the somatic marker hypothesis (Damasio, 1994, 1996) suggests that changes in the visceral states of the body (heart rate, blood pressure, gut, and nervous system activity) act as affective signals that help guide people to adaptive action (Reimann and Bechara, 2010).

Classic research has shown that affect can change perceptions of the probability, or risk, of uncertain events. For instance, reading a newspaper story about the tragic death of a young person leads participants to overestimate the probability of future negative events (e.g., floods, accidents, or diseases) by 74% (Johnson and Tversky, 1983). Specific emotions can also influence explicit likelihood estimates in an emotion-congruent manner: participants induced to feel sadness perceived a greater likelihood of future sad events but not anger-inducing events (DeSteno et al., 2000). Additionally, fearful people make pessimistic risk assessments, while angry people make optimistic risk assessments (Lerner and Keltner, 2001). The risk-as-feelings theory suggests that people use feelings that contain information about risk when making decisions (as opposed to computing the normative “expected utility” of different options; Loewenstein et al., 2001). The authors suggest that uncertainty is represented within two systems: a cognitive system and an emotional system that can agree of conflict with each other (similar to other dual-process theories mentioned above).

In sum, contemporary theories suggest that affect and emotions typically serve an adaptive function of guiding attention, cognition, and action. When not beneficial, affect and emotions can be modified by different regulation strategies (Gross, 2014). While there are a number of theories about the nature of affect and emotion, appraisal theories are particularly relevant for this discussion because they posit that emotions are directly shaped by the perceived certainty of a situation (in addition to other appraisal dimensions).

### The Relationship Between Uncertainty and Affect: Specific Theoretical Accounts

Having established working definitions of uncertainty and affect and surveyed general theories of affect and emotion for key insights about the relationship between these phenomena, we now analyze theoretical accounts that more specifically and explicitly address the influence of uncertainty on affect. Most of these theories describe uncertainty as aversive, and most focus on the relationship between uncertainty and negative affect or emotional states. An exhaustive review of the psychological empirical literature is beyond the scope of the current analysis, and such reviews can be found elsewhere (Carleton, 2016b).

It is also important to note that although we focus here on the psychological literature, researchers in other domains have also studied affect and uncertainty using different methods and from different theoretical perspectives. For example, computational neuroscientists have used modeling techniques have been used to assess psychological, behavioral, and neuropsychological outcomes under conditions of reward and indeterminacy (e.g., Lowe and Ziemke, 2013; Lowe et al., 2017; Babayan et al., 2018; Starkweather et al., 2018). Like past work in other areas, this research has also tended to use variety definitions for affect and uncertainty. Furthermore, to our knowledge this work has not explicitly attempted to analyze the relationship between affect and uncertainty, although we believe it may offer useful insights for further empirical and theoretical work.

#### Behavioral Inhibition System Theory

The influential theory of behavioral inhibition was largely motivated by rodent learning studies and neurophysiological evidence, though it has been extended to anxiety disorders in humans (Gray, 1976; Gray and McNaughton, 2000). This theory specifically posits that novel stimuli, unexpected events, or conflicts between competing behavioral options can all activate the BIS neurological system. The behavioral inhibition system (BIS) is responsible for effectively guiding behavior in these novel or unexpected situations. The BIS provides guidance by suppressing behavior, increasing attention to novel features, and heightening the organism’s arousal which allow the organism to act in an adaptive manner. At the neural level, BIS activation is identified as a 7.7-Hz hippocampal theta response, driven by activity in the septal area.

In Gray and McNaughton’s view, activation of the BIS is tightly coupled to anxiety. They state: “we identify anxiety with activity in the behavioral inhibition system” (Gray and McNaughton, 2000; p. 84). That is, they stipulate that when the BIS activates, the animal or human experiences anxiety. According to this theory, indeterminacy related to novelty or unexpected events activate the behavioral inhibition system, which causes anxiety. The same indeterminacy also likely causes the subjective experience of uncertainty in humans, though it is less clear of what rodents experience. One interpretation of this account is that anxiety is the subjective experience of indeterminacy–just like uncertainty is the awareness of ignorance. The exact link between experienced uncertainty and anxiety is not spelled out, beyond that they are often generated by the very same situations.

One interesting implication of the BIS theory is that the conflict between two positive options would result in BIS activation (e.g., “Should I eat delicious food A or delicious food B”?; Iyengar and Lepper, 2000). Thus, uncertainty could arise in situations that are generally positive. However, the BIS model would still suggest that anxiety would be the affective state produced by multiple appealing options.

#### Uncertainty and Anticipation Model of Anxiety

Building on the BIS model outlined above, the Uncertainty and Anticipation Model of Anxiety (UAMA) theory of uncertainty and anxiety incorporates recent human neuroimaging research (Grupe and Nitschke, 2013). The UAMA model proposes that clinical anxiety disorders are due to heightened expectancies about the probability and cost of future threats. UAMA attributes these effects to changes in five key psychological processes related to uncertainty: (1) changes in the calculation of expected value and aversive prediction error signaling; (2) hypervigilance and increased attention to possible threats; (3) deficient safety learning or an inability to regulate responding in safe situations; (4) subsequently increased cognitive and behavioral avoidance of situations or evidence that contradict negative predictions about the future (which allow the negative predictions to persist); and (5) exaggerated physiological and behavioral reactivity under uncertainty, leading to further avoidance of situations perceived as uncertain. UAMA focuses on clinical anxiety (Grupe and Nitschke, 2013), but the psychological systems are present in healthy populations; therefore, this model could be useful in understanding the link between uncertainty and affect more broadly. However, the link between uncertainty and affect is not the sole focus of the model, and the authors do not explicitly address why uncertainty causes anxiety.

#### Entropy Model of Uncertainty

The Entropy Model of Uncertainty (EMU) model by Hirsh et al. (2012) utilizes the concept of entropy from thermodynamics and information theory to explain the nature and psychological effects of uncertainty. In this model, psychological entropy reflects the amount of uncertainty (i.e., entropy) in a system. This psychological entropy applies to uncertainty about either a perception (“what is that”?) or an action (“what is the right action”?). The authors proposes four tenets in the EMU model: (1) in general, uncertainty is a critical adaptive challenge for organisms, and thus managing uncertainty is important; (2) uncertainty creates conflicts between competing perceptual and behavioral affordances; (3) concrete goals and belief structures can reduce the experience of uncertainty by reducing the set of possible perceptions and actions; and (4) uncertainty is experienced subjectively as anxiety because uncertainty reflects the inability to perceive the world or know which action to take—two evolutionarily fundamental tasks. This theory, however, does not specify exactly why or how uncertainty is associated with anxiety (negative affect) beyond this evolutionary argument. It is descriptive rather than a causal theory that simply characterizes the association between uncertainty and anxiety.

#### Theory of Personal Uncertainty

Personal uncertainty has been described as the aversive feeling that is experienced when one is uncertain about oneself or one’s worldviews (van den Bos, 2009). A central premise is that humans engage in a fundamental process of “sense-making” to understand their lives. Personal uncertainty challenges this “sense-making” process and the meaning people attribute to their lives. Personal uncertainty is experienced as negative and these negative feelings then motivate people to manage their uncertainty (for review see, van den Bos, 2009). One way to manage personal uncertainty is by adhering to cultural values and norms more strongly (e.g., belief in a just world; Lerner, 1980), a strategy that has been demonstrated in empirical studies (van den Bos et al., 2005). After being primed to think about their own personal uncertainty, people become more rigid and closed-minded (McGregor et al., 2001). Additionally, van den Bos (2009) argues that uncertainty may explain some of the effects traditionally attributed to terror management theory (Greenberg et al., 1997). For instance, contemplating death might lead to uncertainty about what will happen after death. van den Bos (2009) contends that although mortality salience may account for various defensive reactions when people are confronted by the threat of mortality, these reactions are also driven by the personal uncertainty that the threat of mortality raises (van den Bos, 2009).

The reactive approach motivation (RAM) theory (McGregor et al., 2009) is compatible with the above work on personal uncertainty and BIS theory (Gray and McNaughton, 2000). In addition to personal uncertainty, the model suggests that anxious uncertainty occurs when a person (or other animal) is caught between conflicting approach and avoidance motivations (McGregor et al., 2010). For example, a hungry mouse which receives a shock when it approaches food is caught in a conflict between a motivation to approach the food and a motivation to avoid the shock. In this theory, “anxious uncertainty” is a term that directly connects affective responses (i.e., anxiety) to uncertainty. RAM theory additionally suggests that ideals function as abstract goals that can guide behavior when lower level goals or actions are blocked (McGregor et al., 2009). Thus, a person can focus on ideals or worldview to help clarify what to do when experiencing anxious uncertainty.

#### Fear of the Unknown Theory

This theory proposes that fear of the unknown is a—and possibly *the*—fundamental fear of human beings (Carleton, 2012, 2016a). A more complete discussion of the empirical background for this theory is available elsewhere (Carleton, 2016a,b), but to summarize, a large body of theoretical, logical, and experimental evidence supports the existence of a fundamental fear of the unknown that appears to be: (1) an emotion; (2) inherent; (3) logically evolutionarily supported; (4) continuously and normally distributed in the population; (5) a logical reduction of higher-order constructs; (6) logically non-derivative and irreducible; (7) able to account for variance in higher-order constructs; and (8) factorially distinct” (Carleton, 2016b, p. 14). Carleton has further suggested that, using an iterative downward arrow approach, other fears are ultimately based on a person perceiving some piece of salient, key, or sufficient information, which ultimately causes an inherent, evolutionarily supported fear response. This suggestion was built upon a proposed contemporary definition for intolerance of uncertainty as, “an individual’s dispositional incapacity to endure the aversive response triggered by the perceived absence of salient, key, or sufficient information, and sustained by the associated perception of uncertainty” (Carleton, 2016a, p. 31). This theoretical proposition and definition allows specific distinctions between stimuli (e.g., an unknown), automatic responses (e.g., a fear response first along the fast pathway and then along the slow pathway; Ledoux, 2000), and engagement with automatic responses (e.g., efforts to endure aversive elements of the automatic response). Carleton (2016a) has also suggested that efforts to predict and control events represent attempts to cope with fear of the unknown and intolerance of uncertainty, and that perceived successes at prediction and control facilitate perceptions of agency and self-efficacy, all of which can progressively reduce fear of the unknown. However, Carleton (2012) has cautioned that attempts to use prediction and control to minimize uncertainty may be less effective for reducing fear and anxiety than increasing one’s individual ability to tolerate uncertainty itself (i.e., to reduce the intensity of an individual’s fundamental fear response to unknowns).

## Theoretical Gaps

We have briefly surveyed a number of psychological theories that have examined the relationship between uncertainty and affect. Some theories from the affective science literature have examined this relationship in a general, indirect manner, mentioning uncertainty incidentally or as an exemplar of stimuli that produce various affective or emotional states (e.g., appraisal theories, Moors et al., 2013). Other theories have examined the relationship more specifically and directly, focusing on uncertainty as a primary cause of affective reactions (e.g., van den Bos, 2009; Hirsh et al., 2012; Grupe and Nitschke, 2013; Carleton, 2016a). These theories differ in how they describe this relationship and what psychological processes they focus on; however, what is more striking is their similarities. They all view uncertainty as a deficit in knowledge that is inherently aversive and results in negative affective states. In general, many theories suggest that animals and humans evolved to perceive or represent the world accurately and that feeling of uncertainty represents the failure of those systems. Uncertainty represents a possibly dangerous situation (“flying blind”) and that evolution has designed the brain to avoid that state at all costs.

In general, while the available literature offers useful insights, important knowledge gaps remain. In particular, none of the theories described here account for the possibility of uncertainty causing positive, rather than negative, affect. This is an important gap, however, given that emerging empirical evidence suggests that uncertainty can indeed be associated with positive affect, and also has more complex, indirect effects on affect. This evidence will now be briefly reviewed.

### Uncertainty and Positive Affect

As described above, most past empirical and theoretical work has focused on negative or undesirable affective responses to uncertainty. Nevertheless, intuitively there are clearly life situations in which affective responses to uncertainty are positive. People watch television shows, movies, sporting events, gamble, and read mystery books in which the outcomes are uncertain—yet these activities are enjoyed. In fact, often these events would be less pleasant if the outcomes are made certain in advance (e.g., spoilers). Currently, a small set of studies provide initial empirical support for the notion that uncertainty can, at times, produce positive affect.

In one study (Kurtz et al., 2007), participants were placed in conditions wherein they were uncertain about which of two gifts they would receive. Participants experiencing uncertainty maintained positive affect longer than those who knew which gift they would receive. Surprisingly, participants who were uncertain about which gift they would receive felt pleasant for a longer period of time than participants who knew they would receive *both* gifts. The same study also found evidence that participants did not accurately forecast how uncertainty would influence their feelings; specifically, participants predicted they would feel most pleasant if they received both gifts. When asked which situation they would prefer to be in, more participants chose the certain condition compared to the uncertain condition, even though participants in the uncertain condition actually felt pleasant longer. To explain these findings, the authors theorized that uncertainty about a pleasant event holds people’s attention, causing them to think more about the possibility of the event which extends their experience of positive affect. For example, uncertainty about which of two gifts might possibly be won leads people to imagine winning the first gift, and to further extend their processing of pleasant scenarios by then imagining winning the second gift. In this way, uncertainty may increase cognitive processing (i.e., two distinct simulations of winning, one for each gift), which then lengthens the experience of positive affect.

### Uncertainty and the Intensification of Affect

Researchers have proposed and tested the “uncertainty intensification” hypothesis, wherein uncertainty during emotional events intensifies incidental affect (Bar-Anan et al., 2009). According to this hypothesis, uncertainty causes negative affect to become more negative and positive affect to become more positive. This hypothesis was tested in four studies that used film clips to induce both positive and negative affect. To manipulate uncertainty, participants repeated phrases conveying certainty (“I see what’s happening”) or uncertainty (“I’m not sure what’s happening”). Participants in the uncertain condition reported more positive feelings toward positive films and more negative feelings toward negative films (as measured by an index of self-reported feelings). The authors also found that participants in the uncertain condition were more curious about the films and that curiosity mediated the relationship between uncertainty and affect. Curiosity, the authors argued, led to greater psychological engagement, which strengthened affective responses to the films and resulted in the intensification of affective states (Bar-Anan et al., 2009).

### Uncertainty and the Dampening of Affect

On the other hand, researchers have also found evidence that uncertainty can dampen or reduce the intensity of affective experiences (van Dijk and Zeelenberg, 2006). In laboratory experiments, participants were asked to imagine themselves in scenarios in which they won various prizes: a CD, dinner for two, or one of the two prizes, but they were uncertain about which prize. Participants who were uncertain about which prize they won were found to experience less intense positive affect compared to participants in the other two conditions. In the second experiment using a similar design, participants were instructed to imagine they lost a lottery ticket that would have won different prizes: a CD, dinner for two, or one of the two prizes (but they were uncertain about which prize). Participants who were uncertain about which prize they lost experienced less intense negative affect associated with the loss. Thus, uncertainty about which prize was won or lost reduced the intensity of associated positive or negative affect—dampening the affective responses. The authors did not propose a specific mechanism for the effect, but raised the possibility that uncertainty might produce “mixed feelings that are difficult to integrate” (van Dijk and Zeelenberg, 2006, p. 175).

### Moderators of the Effects of Uncertainty on Affect: Situational Characteristics

These findings by van Dijk and Zeelenberg (2006) seem to contradict findings from the study by Kurtz et al. (2007), in which uncertainty about a prize led people to experience more positive affect. These seemingly discrepant results might be due to contextual factors, which may moderate the relationship between uncertainty and affect. One potential factor is ecological validity. In the Kurtz et al. (2007) study, the prizes were actually won (participants actually got to take them home), whereas in the van Dijk and Zeelenberg (2006) study, the prizes were hypothetical and participants were asked to *imagine* winning a prize based on a scenario. Whether the prizes were real or hypothetical may have moderated the influence of uncertainty on participants’ emotional experiences.

A moderating role of realism or ecological validity in people’s affective responses to uncertainty has been supported by both empirical evidence and theory. A study by Bar-Anan et al. (2009) found that people do not accurately forecast how uncertainty will influence their experiences of a future gain or loss. Their forecasts often depart from their experienced feelings (consistent with much work on affective forecasting; Wilson and Gilbert, 2005). Theories about negative affective responses to art posit that the perceived reality of a situation influences people’s responses to the situation. People enjoy negative affect in art when there is psychological distance between the event and perceiver, as in obviously contrived situations such as Greek tragedies or horror movies (Menninghaus et al., 2017). Indeed, the authors argue people enjoy those forms of art precisely because they are not real. Uncertainty might function similarly: it is tolerable and even enjoyable in contrived or controlled settings where stakes are low, but intolerable and unpleasant in real-life situations with significant consequences.

Importantly, the studies described here manipulated uncertainty, but did not measure participants’ feelings of uncertainty. Accordingly, clear evidence is still lacking regarding the degree to which participants were truly uncertain in the sense of being consciously aware of their ignorance. The absence of such evidence thus allows for an alternative explanation for the discrepant results of past research—specifically, these discrepant results may result from variation in the levels of uncertainty manipulated in different studies. Lower levels of uncertainty might influence emotions in one direction (intensifying), while higher levels of uncertainty might influence emotions in the opposite direction (dampening). More research is needed to test these and other alternative explanations.

As mentioned previously, situational factors such as the realism of an uncertain stimulus may moderate the effects of uncertainty on affect. But these effects might also be moderated by numerous other situational factors and characteristics of individuals, which have not been fully characterized in past research. One potentially important moderator is the probability of a given outcome. It stands to reason that uncertain outcomes of lower probability, such as getting struck by lightning, would generate weaker affective responses than uncertain outcomes of higher probability, such as contracting the flu during a documented epidemic. Another potentially important situational moderator is the severity of a given outcome. It stands to reason that uncertainty outcomes of lower severity, such as contracting the flu, would generate weaker affective responses than uncertainty about developing cancer. However, we currently lack empirical evidence on these effects.

Characteristics of individuals may also moderate the effects of uncertainty on affect. These include sociodemographic characteristics (e.g., age, sex, and education), as well as health literacy and numeracy, and personality differences. Individuals’ life experiences (e.g., a person’s experience with cancer) will also likely shape responses. Empirical evidence on the moderating effects of these characteristics on the relationship between uncertainty and affect is also lacking, and further research is needed to explore these effects.

### Moderators of the Effects of Uncertainty on Affect: Uncertainty Tolerance

**Figure 2:**
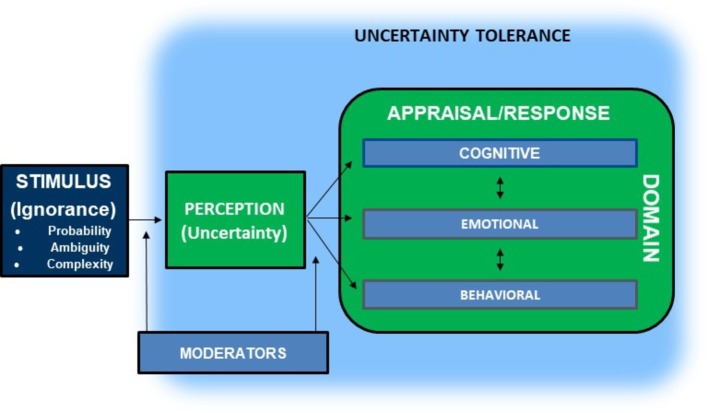
Integrative model of uncertainty tolerance (from Hillen et al., 2017).

A specific characteristic of individuals that has been better studied as a potential moderator of people’s responses to uncertainty is “uncertainty tolerance” (UT). This construct has been described by researchers using various overlapping, inversely-related, and logically equivalent terms (e.g., uncertainty tolerance, uncertainty intolerance, ambiguity tolerance, and ambiguity intolerance) and has also been defined in different ways, although existing definitions include several shared elements. In a recent review of the multi-disciplinary literature on UT (Hillen et al., 2017), we assessed how researchers from various fields have conceptualized UT, either explicitly in published definitions of the construct, or implicitly in published measures used to assess UT. This analysis revealed that UT has been defined with respect to the presence or absence of a wide variety of cognitive, emotional, and behavioral responses to uncertainty—both negative and positive in valence, although mostly negative (Figure 2). Building on previous work and definitions (see Carleton, 2016a), we developed an integrative working definition of UT: “the set of negative and positive psychological responses—cognitive, emotional, and behavioral—provoked by the conscious awareness of ignorance about particular aspects of the world” (p. 70, Hillen et al., 2017).

We argued that UT can be construed as either a state or a stable personality trait that predisposes individuals to specific psychological responses, but that the view of UT as a trait has dominated the literature (Hillen et al., 2017). Available evidence suggests that UT differs among individuals, and UT is associated with other known personality traits including authoritarianism, dogmatism, and openness to experience. Empirical evidence also suggests that differences in UT are associated with various outcomes, including health-related outcomes (Strout et al., 2018). It stands to reason that trait-level differences in UT may moderate the effects of uncertainty on affect.

Persons who report higher UT are more likely to report lower negative affect and higher life satisfaction (Garrison et al., 2017), as well as higher self-esteem and creativity (Pavlova, 2018). Higher UT also appears to be associated with greater risk-taking (Kornilova et al., 2018), whereas lower UT and lower tolerance for ambiguity has been associated with behavioral outcomes indicative of risk aversion (Tsui, 1993; Kornilova et al., 2018). UT appears positively related to adaptability and adaptive readiness (Shamionov, 2017), and more specifically with the ability to cope constructively with chance events (Kim et al., 2016). Persons with greater tolerance for ambiguity may also be more likely to engage in pro-social risk-taking behaviors (Vives and Feldman Hall, 2018). That said, there does not appear to be evidence of a significant relationship between UT and cognitive control functions (e.g., conflict monitoring and attention allocation; Schroeder et al., 2018), suggesting tolerance for uncertainty may indeed be highly malleable (Carleton, 2012, 2016a). Indeed, in a study of medical students, UT was increased as a function of education that specifically focused on increasing tolerance for ambiguity (Taylor et al., 2018).

## Reconciling Findings and Theories: The Central Role of Mental Simulation

As outlined in the previous sections, existing theories offer several useful insights about the relationship between uncertainty and affective and emotional states; however, more work is needed to fully describe the causal relationships between these phenomena. Existing theories do not account for why uncertainty is sometimes associated with positive affect. Rather, existing theories focus on downstream negative consequences of uncertainty and the psychological coping mechanisms people engage in to reduce negative affect (e.g., reaffirming goals and ideals; McGregor et al., 2009; van den Bos, 2009). The critical need moving forward is for an explanatory psychological theory that details the causal pathway from uncertainty to affective responses—both negative and positive. We believe that meeting this critical need will require much more research, both conceptual and empirical, and a synthesis of insights and evidence from multiple disciplinary perspectives. At the same time, we believe that existing insights and evidence make it possible to formulate a tentative, provisional account that can serve as a useful starting point for future work. In the next section, we will outline how mental simulation of possible future events might serve as the core psychological process linking uncertainty to affective and emotional states.

Humans constantly think about possible future events, imagining—or *mentally simulating*—alternative states of reality, such as what it will be like to gossip with friends, confront a co-worker, or commute home by a new route (Seligman et al., 2013; Szpunar et al., 2014; Jing et al., 2016). This *ability to simulate possible states of reality* helps people plan for the future, test out alternative responses, and ultimately cope with stressful situations (Taylor et al., 1998; Moulton and Kosslyn, 2009). In indeterminate situations, mental simulation of possible outcomes is a plausible adaptive strategy. For instance, if a medical test could show disease A or disease B, someone waiting anxiously for the results might first simulate what it would be like to have disease A, and then switch to simulating what it would be like to have disease B. This might help them plan for what would be needed in different possible situations. In this manner uncertainty might invite mental simulation.

We use the term “mental simulation” to cover a broad range of related topics: visual imagery, imagination, mind-wandering, daydreaming, default mode network activity—any kind of self-generated content that involves a simulation of the world. Simulation has emerged as a key, unifying process in cognitive science theories, such as *grounded cognition*—which posits that all mental functions are dependent on simulations (for review, see Barsalou, 2008). Mental simulations are thought to be linked to actions through imagining movements and motor imagery (Jeannerod, 1994). Mental simulations can be effortful and goal oriented, as in mentally rotating objects to determine shape (Shepard and Metzler, 1971). At other times, however, mental simulations can be automatically initiated, as in mind-wandering (Smallwood and Schooler, 2015). Additionally, conscious awareness of mental simulation—in a broad sense covering the range of processes described above—might vary.

Several theories link mental simulation to affective processing. For instance, the somatic marker hypothesis suggests that simulation of bodily states (the reactivation of the “as-if body loop”) play a critical role in decision making and learning about rewards and punishment (Damasio, 1996). Empirically, mental simulations can generate affective and emotional experiences (Lang, 1977, 1979). In fact, affective scientists use mental simulations to intentionally and specifically manipulate affect among participants in experimental studies (e.g., Wright and Mischel, 1982; Strack et al., 1985; Larsen and Ketelaar, 1991). By asking participants to mentally simulate sad or happy events from their own lives, researchers can reliably evoke these emotional states. These affective changes occur with corresponding low-level physiological changes, like fluctuations in heart rate, blood pressure, and electrodermal activity, which demonstrates that the physiological systems involved in affective states are also activated by mental simulations (for review, see Ji et al., 2016).

Thus, mental simulations might represent the critical mechanistic link between uncertainty and affective responses: uncertainty invites simulation of possible situations, and simulation, in turn, generates affective responses. For instance, if someone learns they *might* have cancer, they simulate what they think it would be like to have cancer (e.g., painful symptoms, treatment side-effects, hair loss, and death), which in turn generates negative affective responses.

### The Content of Mental Simulations

If uncertainty induces mental simulations that evoke affective responses, then the nature of the affective responses (positive or negative valence) should depend on the content of the mental simulations. For instance, if uncertainty about playing the lottery induces mental simulations of a positive outcome such as buying a dream home or going on a vacation to Hawaii, then uncertainty will likely lead to pleasant affective feelings. However, if uncertainty about a medical test result induces mental simulations of a negative outcome such as receiving a phone call with a cancer diagnosis, then uncertainty will likely lead to negative affective feelings.

Therefore, the next important question is: What determines the contents of mental simulations? In psychology experiments, the content of mental simulations is often explicitly defined by researchers. For example, researchers instruct participants to simulate particular situations—e.g., remember a time they felt angry or happy. The content of simulation can also be shaped in more subtle ways as well, like describing a dilemma with a win or loss frame (Kahneman and Tversky, 1979).

In everyday lives, however, little is known about what factors determine the content—and ultimately the affective valence—of mental simulations. We do know that minds are active even when people are resting and not given explicit instructions—that is, people’s minds wander (for review see Smallwood and Schooler, 2015). For instance, people spontaneously engage in a variety of mental simulations, including imagining future events, or thinking about an event from the perspectives of other people. This type of mind wandering is quite common (Killingsworth and Gilbert, 2010) and is associated with brain activity in the default network that includes a medial temporal subsystem and medial prefrontal subsystem (Grafton et al., 2007; Buckner et al., 2008; Christoff et al., 2009). Researchers measure activation in this region while participants are instructed to rest or focus on a fixation cross in neuroimaging studies. Understanding exactly why people spontaneously generate particular types of mental simulations in different life circumstances may help elucidate why uncertainty tends to provoke particular affective responses.

### Bias Toward Negative Simulation

If mental simulation is a key process linking uncertainty to affect, then why is uncertainty typically experienced as a negative phenomenon? One possibility is that in situations with indeterminacy about whether a given outcome might be positive or negative, more weight is implicitly given to the potential negative outcome. An attentional bias toward the negative—toward prioritizing negative information—has been observed in many domains and may be a fundamental feature of the mind (for review, see Baumeister et al., 2001). For instance, even when confronted with conflicting visual information in binocular rivalry experiments, the visual system prioritizes faces associated with negative gossip over other faces (Anderson et al., 2011). Additionally, negative beliefs have been shown to influence the perception of food, whereas positive beliefs do not (Anderson and Barrett, 2016). This implicit, biased prioritization of negative stimuli may be an evolutionarily adaptive response, given that the cost of missing a negative threat may be much greater than the cost of missing a positive reward.

This bias toward negative as opposed to positive outcomes and information can be modeled using a framework that combines signal detection (decision making under uncertainty) and economic utility functions (Lynn and Barrett, 2014). Signal detection theory posits that people operate in uncertain situations and must make decisions based on limited, conflicting, or noisy information (signals). For instance, a “signal” might consist of a co-worker’s ambiguous facial expression, which may or may not represent a social threat (e.g., “are they mad at me”?). The co-worker might truly be upset and in need of relationship repair, but may alternatively not be distressed and might be thinking of something else entirely, in which case behavior to repair the relationship might be unneeded or even costly.

Signal detection theory can be used to model the decision-making process as a function of the strength of the signal of potential threats, the perceived base rate of threats, and the perceived costs of missed detection (i.e., missing a threat) and false alarms (false-positive responses to non-existent threats). In situations in which the perceived frequency of threats or the cost of missing them is extremely high, it can be advantageous to liberally classify more signals as threats, by adopting a “zero-miss” strategy: treat any ambiguous stimuli as a threat. For instance, Quigley and Barrett (1999) give the following example of a child who grows up in an abusive household. When faced with uncertainty (i.e., “Is the caregiver angry?”), the child might use a zero-miss strategy that liberally classifies ambiguous expressions as threats. This strategy minimizes missed detections of threat, but simultaneously increases the number of false-positive responses to non-threatening caregiver expressions. This strategy would thus lead to frequent but ultimately unnecessary appeasement behaviors that may be costly from behavioral or social perspectives (Quigley and Barrett, 1999; Lynn and Barrett, 2014).

The adaptive or evolutionary advantage of this tendency to adopt a zero-miss strategy—to categorize uncertain situations as negative by assuming the worst—may explain why individuals most often experience uncertainty as affectively negative (for discussion see Carleton, 2016a). If one is confronted by a dark shadow, it may be more adaptive to act as if there is danger than to act as if there is not. This response may be either inherited or learned through experience, but in any case, simulation is the key link between uncertainty and affective responses. Uncertainty provides an opportunity to predict and simulate negative potential outcomes, and this simulation, in turn, generates negative affective responses.

This link between uncertainty and affect is likely moderated by various factors including trait-level characteristics of individuals. Dispositional optimism and pessimism are two such traits. It stands to reason that uncertainty would result in more aversive affective responses among people who are pessimistic—and thereby predisposed to simulate the worst possible outcomes, and less aversive responses among people who are optimistic—and predisposed to simulate more positive events. To our knowledge, however, little work has tested the factors that moderate and mediate the effects of uncertainty on affective outcomes.

This mental simulation account offers a novel way of understanding the relationship between uncertainty and affect by specifying mental simulation as the causal mechanism linking the two phenomena. Additionally, it suggests new research questions to be explored (see below for more details). This account may have some practical utility as well: if simulation is indeed the causal link between uncertainty and affect, changing what people simulate should change affective responses to uncertainty. More broadly, perspectives from emotion regulation (Gross, 2014) offer theory-based guidance for possible strategies for coping with uncertainty. A more specific mechanistic understanding of the link between uncertainty and affect may help clinicians develop more effective interventions that target the causal pathways in people struggling to cope with uncertainty. We hope this account inspires new conceptual thinking, empirical studies, and practices to help people cope with uncertainty.

## Effects of Uncertainty on Affect: Future Research Needs

Thus far, we have reviewed some empirical findings on the effects of uncertainty on affect, and suggested that mental simulation is a key process linking those phenomena. Uncertainty has been shown to cause both negative and positive affect and can both heighten and dampen existing affective feelings. We have argued that the mental simulation of potential future outcomes is a fundamental psychological process that may account for these effects; variability in affective responses to uncertainty may reflect variability in the content of people’s mental simulations. Clearly, more research is needed to fully specify and test this account against alternative theoretical models. Little is known about baseline individual differences in mental simulations in response to uncertainty. If simulation plays such a key role, it suggests the hypothesis that traits like optimism might moderate the content of simulations and thus affective responses. Moreover, it also suggests the hypothesis that influencing the content of mental simulations should change affective responses to uncertainty. This leads to the possibility that interventions that target simulation might help people regulate affective experience in the face of uncertainty.

Beyond testing hypotheses about the role of simulation in generating affective responses to uncertainty, we believe there are additional research questions that would be fruitful to explore further. We now outline several general questions that need to be answered to advance our understanding of the link between uncertainty and affect.

### Empirical Issues: Mechanisms Linking Uncertainty to Affect

In general, more research is needed to test the different models to elucidate the mechanisms underlying the link between uncertainty and affect. In the next section, we outline suggestions for future work to explore factors that may mediate or moderate the relationship between uncertainty and affect.

#### Mediators

Future research should explore what factors mediate the effects of uncertainty, and what elements of uncertainty represent the “active ingredient” responsible for its main effects. The experience of uncertainty is associated with a number of psychological phenomena: subjective perceptions of a lack of information, as well as other beliefs, attitudes, and judgments; affective feelings; and behavioral responses including information seeking and decision making (for discussion see Hillen et al., 2017). It is possible that any of these features might influence affect and other mental processes. For instance, uncertainty might be associated with attempts to gain more information, or sense making. These processes and not the *feeling* of uncertainty *per se*, might change affective experience (Wilson et al., 2005). Future work is needed to tease apart these components of uncertainty to understand their causal significance.

#### Moderators

More research is also needed to better understand the factors that moderate the effects of uncertainty on affect. Situational context is one potential moderator that needs to be explored in more detail. As described previously, in many situations uncertainty is experienced as unpleasant. However, in some contexts, people enjoy uncertainty (e.g., sports, gambling, movies). Some studies have found that uncertainty can intensify emotions (Bar-Anan et al., 2009) while others found uncertainty dampens emotions (van Dijk and Zeelenberg, 2006). Context appears to be a powerful moderator between uncertainty and affective experiences. For instance, whether people are uncertain about real events or imagined hypothetical scenarios might moderate affective reactions; uncertainty about *imagined* hypothetical scenarios might dampen affect (van Dijk and Zeelenberg, 2006) while uncertainty about *real* events might intensify affect (Bar-Anan et al., 2009).

Future work should also explore how different emotions are related to uncertainty. Past studies have typically explored how uncertainty influences affect and emotions broadly—e.g., intensifying or dampening all emotional and affective experiences (van Dijk and Zeelenberg, 2006; Bar-Anan et al., 2009). It is quite possible, however, that uncertainty has differential effects on particular affective states or specific emotions (e.g., surprise, guilt, disgust). According to appraisal theories (Moors et al., 2013), some emotions have uncertainty as a core appraisal dimension that triggers their activation (e.g., surprise, fear, anxiety—the emotions most commonly studied with uncertainty) while other emotions do not (e.g., guilt, shame, happiness). However, we are unaware of any empirical work has systematically studied the relationship between uncertainty and a broad range of particular affective and emotional states.

Additionally, future work should explore the breadth of variability in how uncertainty relates to affective states. In the evidence reviewed here, uncertainty intensified or dampened affect, but can uncertainty change the valence of an experience from positive to negative (or the other way around)? For instance, are there situations where low uncertainty leads people to experience a negative emotion (e.g., boredom), whereas the introduction of uncertainty into the situation might lead people experience a positive emotion (e.g., interest)? What factors moderate these effects?

Finally, more work should explore the range of individual differences in how uncertainty is experienced. Implicit in the very construct of “uncertainty tolerance” is the assumption that uncertainty is negative, a thing to be tolerated. But are there people at the other end of the spectrum who actually enjoy uncertainty? It is possible that thrill seekers, adventurers, and scientists are drawn to uncertainty? Further, what drives individual differences in uncertainty tolerance? Are they driven by life experience and/or genetic factors?

### Methodological Issues: Measuring Uncertainty and Affect

An important limitation of past experiments examining the effects of uncertainty on affect is that participants’ experience of uncertainty is often experimentally manipulated but not measured. For instance, participants might be placed in the “uncertainty condition” where they are presumed to be uncertain about the outcome of a lottery. While this situation undoubtedly has elements of indeterminacy, the participants’ subjective experience of uncertainty is typically unmeasured (e.g., van Dijk and Zeelenberg, 2006; Kurtz et al., 2007; Bar-Anan et al., 2009). This is an important methodological deficiency given that people may not experience similar levels of uncertainty even when they are in similar situations. This limits our ability to draw firm inferences about the effects of uncertainty on affect. Thus, an important direction for future work is to improve the measurement of uncertainty itself.

A related limitation is the lack of specificity in measures of uncertainty, which have largely treated uncertainty as a monolithic phenomenon. Uncertainty, however, has numerous types that reflect the variety of sources and issues to which it applies (Han et al., 2011). Sources include probability (risk), ambiguity, and complexity, while issues encompass a range of particular problems ranging from scientific to practical to personal in nature (Han et al., 2011). An open empirical question is whether these different types of uncertainty have correspondingly different effects on affect—or lead to different specific emotions (e.g., anger, fear). More research is needed to develop reliable, valid measures that ascertain the variety of uncertainties that arise in life, and to examine their differential effects.

Additionally, there are many domains in which uncertainty is possible (e.g., financial, health, art) and it is not clear how similar or different the experience of uncertainty is in these different domains. This is particularly true for the construct of uncertainty tolerance. Little is known about whether uncertainty tolerance is domain-general or domain-specific.

Currently most measures of uncertainty rely on participant self-report of their experience of uncertainty. While useful, this approach requires introspection and accurate reporting and there may be situations in which these requirements produce inaccurate data. For instance, physicians may be motivated to present themselves as highly skilled and confident (Katz, 2002), which might lead them to underreport their own experience of uncertainty. Other techniques for measuring uncertainty that do not rely on self-reports should be explored and validated. For instance, in cognitive psychology reaction time has been used as a measure of uncertainty or conflict between two options. Mouse-tracking has been used to study decisional conflict as people choose between two or more options, and mouse trajectories might be related to uncertainty (Freeman and Ambady, 2010). Perceptual uncertainty has been linked to eye movements (Brunyé and Gardony, 2017) so eye-tracking may be a promising measure of uncertainty in other domains. The Beads Task has been used to induce uncertainty, and has a behavioral measure that correlates with uncertainty tolerance (Jacoby et al., 2014). Performance on this task might be used as an index of experienced uncertainty.

Finally, as noted in this review, there is evidence of a link between uncertainty and affect, and measures of affect might thus be used to draw inferences about experienced uncertainty. For instance, in contexts where affect has been shown to result from uncertainty (e.g., gambling), peripheral psychophysiology might yield information about experienced uncertainty. For example, in the Iowa Gambling task, participants choose between decks cards that could result in wins or losses (Bechara et al., 2005). Participants reliably have skin conductance responses when they are uncertain about the outcome of their gamble (Bechara et al., 2005). Researchers have traditionally used these skin conductance measures in this context to study affective responses to these gambles. However, those affective responses might be due in part to experienced uncertainty. Further work would be needed to explore the utility of these measures and experimental paradigms for studying uncertainty specifically.

### Conceptual Issues: Uncertainty, Ignorance, and Consciousness

Uncertainty is ubiquitous, touching almost every aspect of our lives. As such, uncertainty has been studied from numerous disciplinary perspectives, including information theory, psychology, judgment and decision making, and economics, all of which have conceptualized uncertainty using different terminology and construed its nature and effects using different conceptual and methodological tools (Smithson, 1989; Djulbegovic et al., 2011). The result has been a vast but often unconnected literature with both similar insights that have been expressed in different ways and different insights that have been expressed in similar ways. Theoretical and empirical findings from different fields have not been integrated in coherent fashion. Developing and advancing the science of uncertainty will require focused efforts to connect these findings.

#### Levels of Uncertainty and Consciousness

A particularly important and unresolved conceptual problem relates to whether uncertainty represents a conscious or unconscious state. In line with our previous work, here we have defined uncertainty as the conscious awareness of ignorance (Han et al., 2011). However, we acknowledge that much cognition occurs unconsciously, automatically, and rapidly, and that no bright lines separate conscious *vs*. unconscious, deliberate *vs*. automatic, slow *vs*. fast cognitions. Consciousness is thus appropriately construed as a continuous rather than categorical phenomenon (Pessoa et al., 2005; Rouder and Morey, 2009; Tamietto et al., 2015). Cognitions become more unconscious, automatic, and rapid as expertise is developed (Logan, 1988), and the same may be true for mental simulations provoked by uncertainty.

Unconscious “uncertainty” has become a major theme in cognitive science, and recent work construes the brain as a “prediction machine” (Friston, 2010; Clark, 2013). The perceptual system in particular has to make predictions based on noisy incoming signals of unknown significance. For instance, light striking the retina can be reflected off a number of sources, leading to perceptual uncertainty about exactly what objects are in the environment. According to predictive theories such as Clark’s, the brain perceives stimuli by matching incoming “bottom-up” sensory signals from the world with “top-down” predictions from the brain. When predictions are incorrect, neural error signals feedback to perceptual brain areas, enabling the brain to tune and calibrate future predictions. This feedback system, called predictive coding, enables the brain to reduce mismatches between the actual world and mental representations of it, fine-tuning an exquisite prediction machine to an ever-changing environment.

This emerging theoretical perspective construes responding to perceptual uncertainty as *the* fundamental task and challenge of the brain. The view of the mind as a prediction machine expands and refines our understanding of uncertainty as a “conscious” experience, but also calls into question the conceptual distinction between ignorance and uncertainty. If uncertainty can be unconscious as well as conscious, then the distinction between ignorance and uncertainty becomes obliterated.

We believe this conceptual equation goes too far—that there are qualitatively discrete levels of consciousness of ignorance, and defensible reasons for distinguishing between them. Even if unconscious uncertainty (what we call “ignorance”) is psychologically consequential at an automatic and perceptual or sensorimotor level, there still exists an important, categorically discrete realm of everyday human experience involving conscious deliberation and awareness of ignorance (what we call “uncertainty”), and this conscious awareness is also psychologically consequential. The vast body of research to date has shown that moving people from unconscious to conscious ignorance clearly has cognitive, emotional, and behavioral effects. The existence of these effects provides empirical justification for the distinction between conscious and unconscious ignorance, and we believe the respective terms “uncertainty” and “ignorance” provide a useful short-hand or linguistic representation of this distinction.

More research is needed to understand the affective and emotional effects of uncertainty existing at different levels of conscious awareness, ranging from complete unconsciousness (pure ignorance) to complete consciousness of ignorance (pure uncertainty). Additionally, more work is need to understand how automatic and rapid—vs. deliberate and slow—people’s cognitive and affective responses to uncertainty are in different life situations.

#### Applied Issues: Improving Affective Responses to Uncertainty

From a practical standpoint, people struggle with uncertainty in their lives. Uncertainty can lead to suboptimal decision making, negative affect, diminished well-being, and psychopathology (Gray and McNaughton, 2000; Rabin and Thaler, 2001; McGregor et al., 2009; van den Bos, 2009; Hirsh et al., 2012; Grupe and Nitschke, 2013; Shihata et al., 2016; Carleton, 2016a; Strout et al., 2018; Vives and Feldman Hall, 2018). Addressing these problems requires applied research aimed at improving people’s ability to regulate and cope with the negative emotional effects of uncertainty.

The ability to regulate affective/emotional responses has been increasingly recognized as an important factor in health psychology (for discussion, see DeSteno et al., 2013), and emotion regulation may play a critical role in people’s ability to cope with uncertainty. When someone experiences negative affect resulting from uncertainty, it should be possible to use various regulatory strategies to reduce the unpleasant experience. For instance, someone who has been diagnosed with early stage cancer might suffer from anxiety while waiting for further medical tests to be conducted. One strategy might be to re-appraise the situation: “Uncertainty about what type of cancer I have means there is a chance my cancer is benign–I’ll focus on that possibility.” They could also use distraction: “I will keep busy to try to not think about the fact that my cancer could progress.” These strategies might aim to both diminish negative feelings and enhance positive feelings.

In addition to strategies aimed at regulating one’s affective responses to uncertainty, people might also adopt strategies aimed at reducing uncertainty directly. These strategies can take many forms, from conducting another test to clarify a diagnosis to finding a specialist for a second opinion. However, these actions also raise the possibility of obtaining conflicting information—for example, differing opinions among experts or conflicting test results—which can generate greater uncertainty.

People may also cope with uncertainty in pathological ways (Grupe and Nitschke, 2013; Carleton, 2016a). For example, individuals with obsessive compulsive disorder might use repeated checking behaviors to manage their uncertainty (Tolin et al., 2003). Similarly, individuals with generalized anxiety disorder might engage in repeated worrying to manage their perceptions of uncertainty (Dugas et al., 1997; Davey and Wells, 2006). The full cause-effect pathway for the relationship between coping with uncertainty and pathology has not been definitively resolved (Shihata et al., 2016); however, there is substantial evidence that increased uncertainty tolerance produces self-reported and behavioral reductions in pathology (Hewitt et al., 2009; Barlow et al., 2011; Farchione et al., 2012; Mahoney and McEvoy, 2012; Boswell et al., 2013; Cuijpers et al., 2014; McEvoy and Erceg-Hurn, 2015). This suggests uncertainty tolerance and associated coping may be important etiological factors (Boswell et al., 2013; Carleton, 2016a). In any case, understanding the ways people regulate and ultimately cope with uncertainty is an important future research direction. Greater understanding of the diversity of regulatory and coping strategies and the outcomes of these strategies can then inform the development of interventions to help people cope more effectively with uncertainty in their lives.

#### Reversing the Causal Arrow: Can Affect Influence Uncertainty?

In this paper we have focused on uncertainty causing affective feelings, but causality in the opposite direction might be possible too. That is, can affective feeling influence the experience of uncertainty? Do people experiencing particular emotions have different experiences of uncertainty? We are not aware of any data or theories showing this directly, but as described earlier there is a vast literature describing how affect and emotions influence perceptions of risk (Johnson and Tversky, 1983; DeSteno et al., 2000; Lerner and Keltner, 2001; Loewenstein et al., 2001) and decisions (Schwarz and Clore, 1983; Damasio, 1994, 1996; Slovic et al., 2007) in situations of complexity, ambiguity, and indeterminacy. Thus, affect and emotions seem to influence the mind in contexts where uncertainty is also present. More research is needed to directly test whether affect can directly influence uncertainty, and whether there is a bi-directional relationship between uncertainty and affect. Future theoretical work should attempt to integrate both causal directions into one theory: how uncertainty influences affect and how affect influences uncertainty.

## Conclusions

The goal of this article was to briefly review and synthesize the literature on the relationship between uncertainty and affect. Although most empirical and theoretical research to date has focused on the negative effects of uncertainty on affect (Gray and McNaughton, 2000; McGregor et al., 2009; van den Bos, 2009; Hirsh et al., 2012; Grupe and Nitschke, 2013; Carleton, 2016a), there is some experimental evidence suggesting that uncertainty has positive effects (Kurtz et al., 2007) and also intensifies (Bar-Anan et al., 2009) or dampens affective feelings (van Dijk and Zeelenberg, 2006). The obvious question is why uncertainty leads to these different outcomes and what factors moderate and mediate its effects? Existing theoretical frameworks do not focus explicitly on the relationship between uncertainty and affect, and are descriptive rather than explanatory. We suggest that uncertainty influences affective states by prompting the mental simulation of possible future outcomes. Additionally, people have a propensity to primarily simulate negative outcomes, which, in turn, tends to generate negative affect. We also propose the existence of several important moderators of this process, including context and other situation factors, as well as individual differences such as uncertainty tolerance. Our synthesis also highlights how negative responses to uncertainty may also be controlled by emotion regulation strategies. Finally, we offer hypotheses generated by our approach, highlight important knowledge gaps, and promising areas for future research, both empirical and conceptual, to improve our understanding of the relationship between uncertainty and affect.

## Author Contributions

All authors listed have made a substantial, direct and intellectual contribution to the work, and approved it for publication.

### Conflict of Interest

The authors declare that the research was conducted in the absence of any commercial or financial relationships that could be construed as a potential conflict of interest.
